# Inhibition of a sulfate reducing bacterium, *Desulfovibrio marinisediminis* GSR3, by biosynthesized copper oxide nanoparticles

**DOI:** 10.1007/s13205-016-0403-0

**Published:** 2016-03-01

**Authors:** Kiana Alasvand Zarasvand, V. Ravishankar Rai

**Affiliations:** Department of Studies in Microbiology, University of Mysore, Mysore, 570006 India

**Keywords:** Corrosion, SRB, Biosynthesis, Nanoparticles, Copper oxide

## Abstract

To control the severe problem of microbiologically influenced corrosion, industries require highly potent antibacterial agent which can inhibit the growth of bacteria on man-made surfaces. This need drove the research towards the synthesis of nanoscale antimicrobial compounds. We, therefore, screened several bacteria for the biosynthesis of copper/copper compound nanoparticles which could inhibit the growth of *Desulfovibrio marinisediminis*, a sulfate reducing bacterium. Supernatant of thirty bacteria isolated from the biofilm formed on ship hull was mixed with 1 mM CuCl_2_ solution at room temperature. Eight bacterial strains, whose mixtures exhibited colour change, were selected for antimicrobial test. One nanoparticle which has been biosynthesized by *Shewanella indica* inhibited the growth of *D. marinisediminis*. Characterization of this particle by UV–visible spectrophotometer, XRD, TEM, DLS and FTIR showed that the particle is polydisperse CuO nanoparticle with average size of 400 nm.

## Introduction

Paint and coating industries get the benefit by the use of NP to reduce corrosion. Addition of NP modifies morphology, conductivity and different physical properties of coats and provides superior resistance against metal corrosion (Montemor 2014). Microorganisms have an important role in corrosion. They cause rapid and severe type of corrosion failure which is well documented by many industries including shipping industry, offshore oil and gas production, power plants and coastal industrial plants (Licina and Cubicciotti 1989; Bodtker et al. 2008; Inbakandan et al. 2010). To manage microbial corrosion, nano-scale materials such as nanosilver and nanotitanium dioxide with potent antimicrobial activity are used to inhibit the microbial growth (Yu et al. 2003; Naik and Kowshik 2014). Copper and copper oxides are other nanoparticles possessing high toxicity. Their toxic effect found to be due to generation of reactive oxygen species, lipid peroxidation, protein oxidation and DNA degradation in the bacterial cells (Chatterjee et al. 2014).

These compounds have been synthesized by physical and chemical methods including thermal reduction, vacuum vapor deposition, microwave irradiation, chemical reduction, and laser ablation methods (Liu and Bando 2003; Zhao et al. 2004; Tilaki and Mahdavi 2007; Dang et al. 2011; Sohrabnezhad et al. 2014). In these methods, hazardous chemicals are being used for the synthesis of Cu and CuONP.

A growing need for simple and viable alternative to toxic chemical and/or physical methods drives researcher to synthesize NP by environment-friendly techniques. There are many reports on antimicrobial activity of available biosynthesized nanoparticles (NP) and accessible bacteria rather than specific ones such as sulfate reducing bacteria (SRB) (Demurtas and Perry 2014; Haider et al. 2014; Khatoon et al. 2015; Razi et al. 2015). SRB are anaerobic bacteria reducing sulfate to corrosive sulfide causing severe corrosion of metals. Many industries including maritime industries experience corrosion problems due to the presence of SRB (Wade et al. 2011). Since the antimicrobial activities of NP are dependent on the physiochemical properties of NP and type of bacteria, in the present study, we screened biosynthesized CuONP which have antimicrobial activity against *Desulfovibrio marinisediminis* GSR3, a corrosion causing bacteria isolated from the ship hull.

## Materials and methods

### Bacterial isolation and growth condition

Bacterial biofilm formed on corroded metal pieces from underwater hull of a fishing vessel in Goa, India, was harvested and used for the study. Each metal piece was washed three times with sterile phosphate buffer solution to remove any loosely attached bacteria. The remaining sessile bacteria were scraped off with sterile scalpel and then transferred to 100 ml sterile glass container filled with Zobell marine broth 2216 (Himedia, India). The bottle was incubated at 27 °C for 48 h. After successful growth, bacterial colonies were purified and subcultured in Luria–bertani broth for further investigation. *D. marinisediminis* GSR3 (KR303707) isolated from the same ship hull was used as test microorganism. This bacterium was cultivated in 15 ml screw cap tube containing Postgate’s B medium (5 ml 70 % sodium lactate, 0.5 g KH_2_PO_4_, 1.0 g NH_4_Cl, 1.0 g Na_2_SO_4_, 2.0 g MgSO_4_·7H_2_O, 1.0 g yeast extract, 0.1 g CaCl_2_·2H_2_O, 0.5 g FeSO_4_·7H_2_O and 20 g NaCl in 1L of distilled water) (pH 7.5–8.0) and sodium thioglycolate (0.1 g) and vitamin C (0.1 g) as reducing agents.

### Biosynthesis of nanoparticles

The isolated bacteria were inoculated in 100 ml Erlenmeyer flask containing 50 ml of Zobell marine broth and incubated for 48 h at 27 °C. After incubation, the cell-free supernatant was recovered by centrifugation at 8000 rpm for 10 min. About 40 ml of the supernatant was added to the 250 ml conical flask containing 40 ml CuCl_2_ solution (1 mM). The flasks were incubated at 27 °C. When the solution colour changed to yellowish-brown, the synthesized NP were isolated by centrifugation and repeated washing with sterile distilled water to remove the salt. Sterile Zobell marine broth mixed with CuCl_2_ was used as negative control.

### Determination of antimicrobial activity of biosynthesized NP

To determine the antimicrobial activity of biosynthesized NP on SRB, a density of bacteria equal to 0.5 Mcfarland was used to test the susceptibility. The aliquots (450 μl) were dispensed in sterile microcentrifuge tubes and 50 μl of NP extracts was added. Microcentrifuge tubes were incubated anaerobically for 2 h at 27 °C. To check the effectiveness of treatment, bacterial numbers were enumerated by drop plate method (Chen et al. 2003). Polymyxin B (100 μg/ml) was used as a positive control. *S. indica* supernatant and SRB culture without any treatment were used as negative control.

### Determination of minimum inhibitory concentration (MIC)

Minimum inhibitory concentration of CuONP was determined by the broth microdilution method. Twofold serial dilutions of nanoparticles were prepared with sterile Postagte’s B broth. The dilutions were prepared at concentration equal to twice the desired final concentration. Further, 100 μl of NP solution was transferred into each well of 96-well plate and 100 μl culture of the test bacteria was inoculated to the wells to obtain the total volume of 200 μl. The final concentration of nanoparticles in the wells ranged from 0.7 to 200 μg/ml. After 7 days of anaerobic incubation at 27 °C, MIC was determined as the lowest concentration of nanoparticles which did not allow blackening of the medium.

### Characterization of NP

Bioreduction of copper ions in aqueous solution was initially monitored by visual observation. Further, to confirm the biosynthesis of CuONP, characterization was done by UV–Vis spectroscopy, XRD, TEM, DLS and FTIR. UV–visible spectroscopy analysis of colloidal CuONP was carried out on Multiskan Spectrum spectrophotometer (Thermo scientific, Germany) at the wavelength range of 300–700 nm. Powder XRD patterns were recorded on RigakuminiFlex 11 diffractometer at 30 kV, 15 mA for Cu Ka radiation (*k* = 1.5406 A°) with a 2θ scanning range of 6–80° at 5 min^−1^. Dynamic Light Scattering (Zetasizer, Malvern) was used to determine the size distribution of particles in the colloidal solution. The morphology, dispersity and of CuONP were studied by TEM analysis (Tecnai G2 spirit BioTWIN, 20–120 kv, Netherland). Sample for TEM studies was prepared by adding a drop of NP suspension onto a carbon coated 200 mesh copper grid and allowed to dry at room temperature prior to examination. Functional groups present in biosynthesized NP were analyzed by FTIR. Fine freeze-dried powder of CuONP were used for the analysis, and the FTIR spectra were obtained using a spectrophotometer (FTIR, Jasco-460 plus) in the spectral region of 400–4000 cm^−1^ using a resolution of 4 cm^−1^.

### Molecular identification of screened bacteria

Bacteria which synthesized NP with inhibitory activity were identified by the use of 16S rRNA gene sequences. 16S rDNA locus was amplified by universal primer pairs, 27F and 1492 R (Lane 1991). The PCR condition included an initial denaturation of 94 °C for 7 min followed by 35 cycles of 94 °C for 1 min, 56 °C for 1 min, 72 °C for 1 min with a final extension at 72 °C for 7 min. The PCR product was sequenced by Sanger’s dideoxynucleotide sequencing method and the obtained sequence was submitted to the GenBank, NCBI (National Center for Biotechnology Information).

## Results and discussion

Industries use chemicals, mainly biocides to mitigate microbial corrosion. However, due to the emergence of bacterial resistance, they tend to look for alternative antimicrobial agents. Interest in the application of nano-sized antimicrobial compounds is on the increase. Nano-sized materials have higher antimicrobial properties as compared to the bulky ones because by decreasing the dimension of the compounds, the surface to volume ratio will get increased and this property augments their interaction with bacterial cell membrane (Hajipour et al. 2012). Though these compounds have good antimicrobial activity, they can be synthesized by Microorganism. Bacteria can very well be used as nanofactories. Biosynthesis of nanoparticles by microorganisms has been attributed to energy production, special functions and detoxification of heavy metals (Krumov et al. 2009). Ramanathan et al. (2013) hypothesised that *Morganella* sp., a silver-resistant bacterium with ability to biosynthesis silver NP, is able to produce copper NP because proteins responsible for bacterial resistant to silver and copper were highly similar. This bacterium was able to produce spherical copper/copper oxide NP with the size of 7–15 nm. Although there are some reports regarding the biosynthesis of copper and its oxide by *Escherichia coli, Morganella morganii* and *Pseudomonas stutzeri,* the exploitation of bacteria as biological resource material for synthesis of Copper NP needs further explorations (Singh et al. 2010; Varshney et al. 2010; Ramanathan et al. 2013). In the present study, we isolated bacteria from the biofilm (an environment where vast diversity of bacteria live in packed community) formed on corroded ship hull which was covered with paint containing copper. Initially, bacteria have been screened for the synthesis of nanoparticles. After adding bacterial supernatant with CuCl_2_ solution, colour change from light yellow to light brown was observed in eight mixtures. This colour change is the primary indicator for the synthesis of CuONP.

**Fig. 1:**
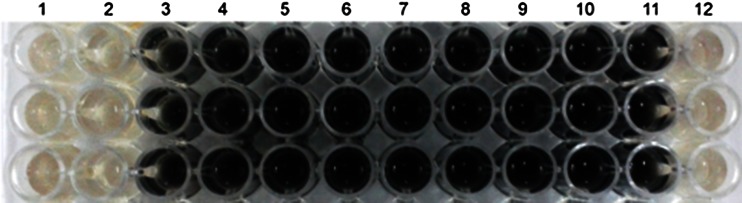
Minimum inhibitory concentration of biosynthesized CuONP against *D. marinisediminis* (wells NO. 1–10 contains 200, 100, 50, 25, 12.5, 6.25, 3.1, 1.5 and 0.75 μg/ml CuONP and wells NO. 11 and 12 are negative and positive control)

After preliminary screening, the selected mixtures were used for antimicrobial test against *D. marinisediminis.* Among the eight mixtures, only one exhibits antimicrobial activity against SRB. Treatment of bacterial solution with CuONP reduced bacterial number from 9 × 10^6^ CFU/ml (Number of bacteria in untreated sample) to 4 × 10^3^ CFU/ml (Table 1). Minimum inhibitory concentration of NP, which is defined as the lowest concentration at which there is no blackening of media, was found to be 100 μg/ml concentration (Fig. 1).

**Table 1:** Antimicrobial activity of CuONP against *D*. *marinisediminis*

	Number of bacteria (CFU/ml)	
Test	8 × 10^3^	
Positive control	3 × 10^5^	
Negative control	1	9 × 10^6^
	2	2 *×* 10^6^

+, Polymyxin B; −, (1) No treatment (2) *S. indica* supernatant

CuONP has potent antimicrobial activity for various bacteria such as *Escherichia coli, Bacillus subtilis, Streptococcus Mutans, Pseudomonas aeruginosa* and *Staphylococcus aureus* (Perelshtein et al. 2009; Azam et al. 2012; Padil and Cernik 2013; Ramazanzadeh et al. 2015). However, as it has been shown by studies, the antimicrobial activity of NP is shape and size dependent (Pal et al. 2007; Ajitha et al. 2015). Padil and Cernik (2013) studied the antimicrobial activity of 4 and 7 nm CuONP which has been synthesized by gum karaya. They observed better activity of smaller particle against both Gram-positive and Gram-negative bacteria. Since the other seven mixtures did not show any antimicrobial effect on *D. marinisediminis*, they have not been characterized; however, we considered that their failure to inhibit the bacteria is due to their inappropriate size or shape.

**Fig. 2:**
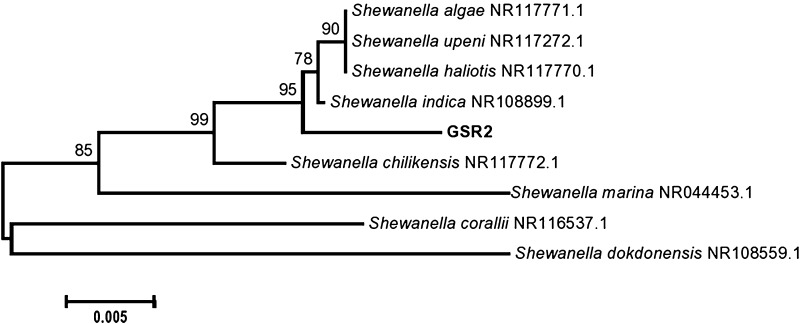
Phylogenetic tree of partial 16S rDNA obtained from *Shewanella indica* subsp. GSR2

Molecular characterization of strain responsible for synthesis of bioactive nanoparticles revealed that the isolate belonged to *Shewanella indica* subsp. GSR2 (accession number: KR303706) (Fig. 2). *Shewanella* can change the oxidation states of metals. The ability of *Shewanella* spp for the biosynthesis of NP; Palladium, gold and iron oxide has been proved previously (Konishi et al. 2007; Bose et al. 2009; De Corte et al. 2011). However, there is no earlier report regarding the ability of *S. indica* to biosynthesis copper or copper compound nanoparticle.

**Fig. 3:**
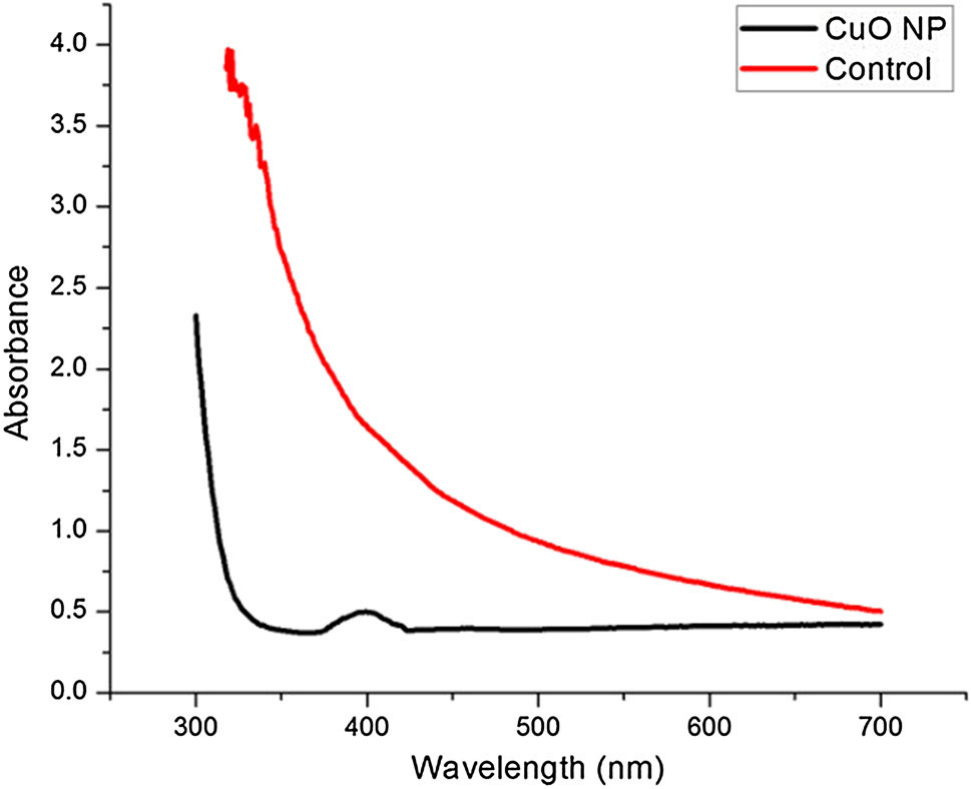
UV–visible spectra of CuO nanoparticles synthesized by supernatant of *S. indica*

**Fig. 4:**
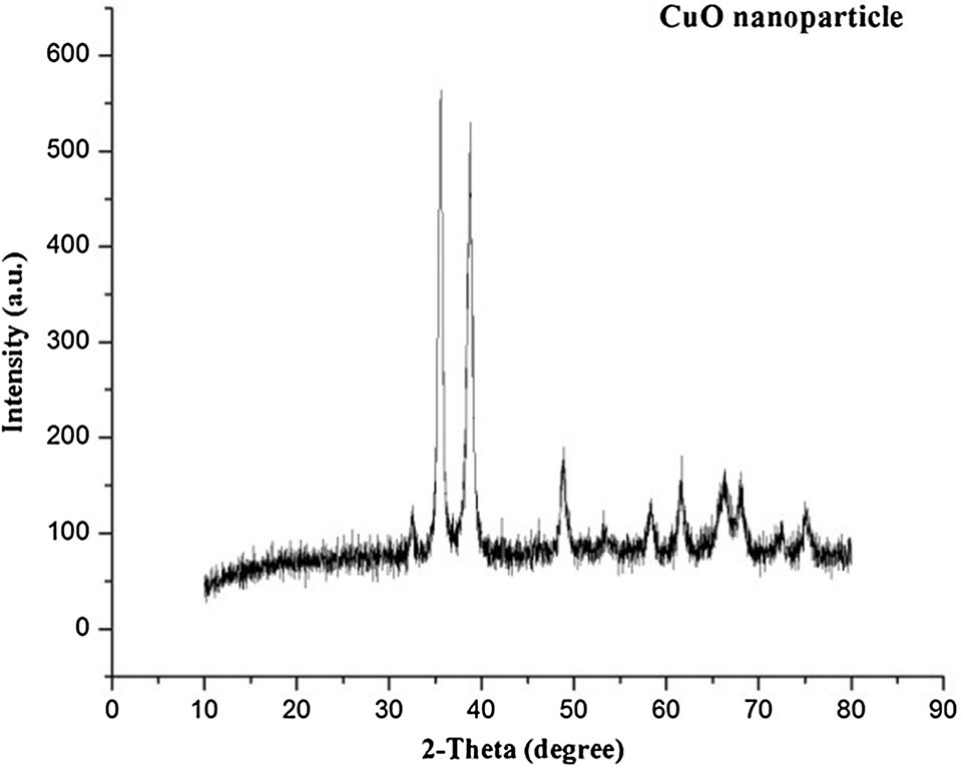
X-ray diffraction pattern of copper oxide nanoparticles synthesized by supernatant of *S. indica*

The biosynthesized NP were characterized using UV–visible spectroscopy, XRD, TEM, DLS and FTIR. The basic characterization of compound by UV–visible spectroscopy showed formation of broad absorbance band around 399 nm which suggests the formation of CuONP (Khanehzaei et al. 2014) (Fig. 3). This result has been confirmed by XRD analysis. The diffraction peaks of sample positioned with 2θ value of 35.5, 38.7, 48.7, 58.1, 61.5, 65.9 were assigned to −110, 111, −202, 202, 113, 022 planes. These planes were matched with the value of crystal CuONP (JCPDS card no. 89-5895) (Fig. 4).

**Fig. 5:**
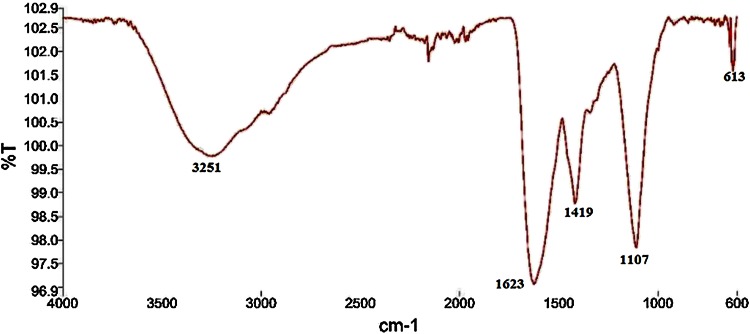
FTIR spectra of CuO nanoparticles synthesized from supernatant of *S. indica*

**Fig. 6:**
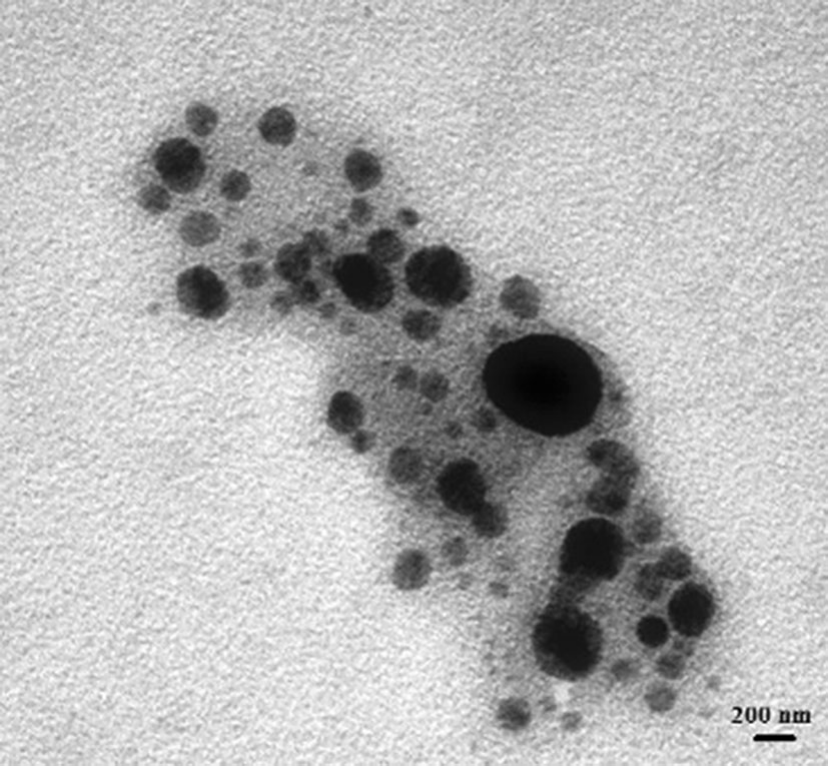
TEM image of biosynthesized CuO nanoparticles

**Fig. 7:**
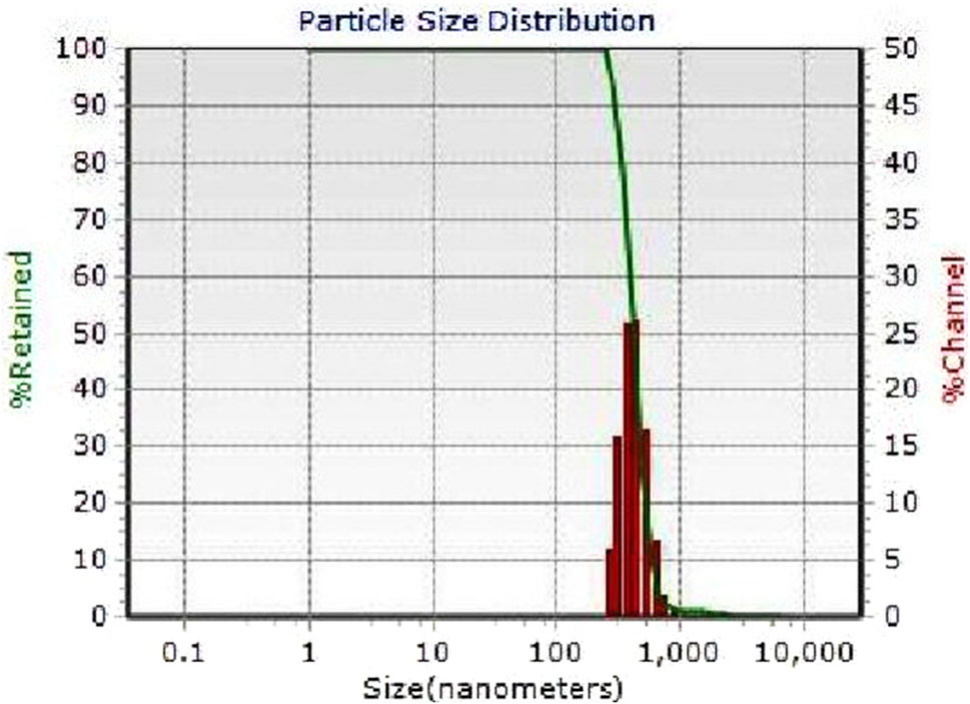
Size distribution of CuO nanoparticles synthesized from supernatant of *S. indic*

The presence of bioactive compound in bacterial supernatant has been proved by FTIR spectroscopy (Fig. 5). The spectral analysis revealed the presence of vibration bands at 1623, 1419, 1107, 613 and an intense broad band at 3251 cm^−1^. The band at 3251 cm^−1^ is due to hydroxyl functional groups (O–H stretching) of alcohol/phenol derivatives (Yugandhar and Savithramma 2015). The band at 1419 cm^−1^ corresponds to the carboxylic group and the band at 613 cm^−1^ is due to C–H stretching. The bands at 1623 and 1107 cm^−1^, respectively, correspond to the –N–H stretch and C–O–C stretch vibrations in amide linkages (amide I and amide II), indicating the involvement of protein/peptide in encapping the nanoparticles (Coates 2000; Salehizadeh et al. 2012). Formation of protein coating on the NP surface prevents agglomeration of metal NP and also helps in their stabilization. The TEM micrograph shown in Fig. 6 clearly demonstrates the formation of spherical particles with different sizes between 150 and 600 nm. In contribution with TEM results, the DLS analysis also showed the formation of polydispersed particles with average size of 400 nm (Fig. 7).

## Conclusion

Use of nano-antimicrobial compound to control biocorrosion is an attractive option for industry because of its high biocidal activity. These particles could be synthesized by physical, chemical and biological methods. As the biological method is eco-friendly, it has got profound interest compared to other methods. In the present study, extracellular synthesis of polydispersed CuONP with average size of 400 nm by *S. indica* strain has been reported. These NP have the ability to inhibit the growth of metal corrosion causing bacteria, *D. marinisedimins*. With our finding and earlier report on ability of *Shewanella* biofilm in inhibition of copper corrosion, it seems reasonable to protect copper from MIC by *Shewanella* live biofilm (Kusa et al. 2006). The advantage of this process as compared to adding nanoparticle to paint is that the nanoparticles do not leach out of biofilm and kill only those bacteria that contact the biofilm.
